# The *Laetiporus sulphureus* Fermented Product Enhances the Antioxidant Status, Intestinal Tight Junction, and Morphology of Broiler Chickens

**DOI:** 10.3390/ani11010149

**Published:** 2021-01-11

**Authors:** Wei Chih Lin, Tzu Tai Lee

**Affiliations:** 1 Department of Animal Science, National Chung Hsing University, Taichung 402, Taiwan; waynezi2227738@gmail.com; 2 The iEGG and Animal Biotechnology Center, National Chung Hsing University, Taichung 402, Taiwan

**Keywords:** *Laetiporus sulphureus*, broiler chickens, antioxidant, tight junction, intestinal morphology

## Abstract

**Simple Summary:**

This study investigated the effects of the *Laetiporus sulphureus* fermented product (FL) as a feed supplement on antioxidant activities, intestinal Tight Junction (TJ) mRNA expression, and the intestinal morphology of broiler chickens. FL supplementation could potentially enhance the feed conversion ratio in broilers by improving their antioxidative status, TJ mRNA expression, and intestinal morphology. Broilers supplemented with 5% FL exhibited the best overall results on improving antioxidant status, TJ mRNA expression, and intestinal morphology.

**Abstract:**

The *Laetiporus* sp. is a fungal species that is traditionally used for medicinal purposes. This study investigated the effects of the *Laetiporus sulphureus* fermented product (FL) as a feed supplementation on the antioxidant activities, the intestinal Tight Junction (TJ) mRNA expression, and the intestinal morphology of broiler chickens. Four-hundred one-day-old male broilers (Ross 308) were randomly allocated to five experimental diets: (1) a corn-soybean meal basal diet (control), (2) a basal diet replaced with 5% Wheat Bran (5% WB), (3) a basal diet replaced with 10% WB (10% WB), (4) a basal diet replaced with 5% FL (5% FL), and (5) a basal diet replaced with 10% FL (10% FL). The FL-supplemented groups exhibited a better feed conversion ratio in the overall experimental period compared to the WB and control groups. The serum antioxidant profiles of 35-day-old broilers showed that, compared to the control and 10% WB groups, the 5% FL supplementation group had a significantly increased superoxide dismutase activity, while it down-regulated the concentration of malondialdehyde in the serum (*p* < 0.05). The assessment of selected antioxidant gene expression showed that the 5% FL group significantly elevated heme oxygenase-1 and nuclear factor erythroid 2–related factor 2 expression, compared to the control and WB groups (*p* < 0.05). Furthermore, both of the FL supplemented groups had a significantly higher expression of glutathione peroxidase and catalase, compared to that of the WB and control groups in the jejunum (*p* < 0.05). The TJ mRNA expression in the jejunum showed that 5% FL significantly elevated the zonula occludens-1, claudin-1, and mucin-2 expression (*p* < 0.05), while 5% and 10% FL supplementation significantly improved *OCLN* expression in both the jejunum and ileum, compared to control group (*p* < 0.05). The intestinal morphology of 35-day-old broilers showed that a 5% FL supplementation significantly increased the villus height in the ileum and jejunum, compared to the WB and control groups (*p* < 0.05). Moreover, the 5% and 10% FL supplementation groups had a significantly higher villi:crypt ratio in the ileum, compared to the WB and control groups (*p* < 0.05). To conclude, FL supplementation improved the antioxidative status, the TJ mRNA expression, and the intestinal morphology, and it was accompanied by a lowered feed conversion ratio in broilers. Finally, 5% supplementation had the overall best results in improving the antioxidant status, TJ mRNA expression, and intestinal morphology of broilers.

## 1. Introduction

Oxidative damage is a critical problem in the poultry industry. It occurs when exogenously- and/or endogenously-produced Reactive Oxygen Species (ROS) exceed the antioxidant capacity of cells and extracellular spaces, causing the disruption of the normal cellular function by influencing the gene expression and signal transduction [1]. World poultry populations are mainly located in tropical and subtropical regions, where heat stress is a main concern, as it negatively influences the antioxidant status, which is reflected by the increased serum lipid peroxidation and reduced plasma concentrations of antioxidants [2]. These stress factors affect the health status of poultry and the safety of poultry products, while they adversely influence the intestinal oxidative status and disrupt the normal function of enterocytes, causing an abnormality in nutrient absorption and diseases [3]. As a consequence of suppressed nutrient absorption, the production indexes (such as feed efficiency and survival rate) are impaired, leading to heavy economic losses to the poultry industry [4].

Tight Junctions (TJs) are intercellular junctional complexes that maintain epithelial cells adherent to each other and guarantee the paracellular transportation of nutrients, forming a barrier between the lumen and host to prevent bacterial translocation [3]. The disruption of TJ impaired the normal intestinal function causing leaky gut that compromises the absorption of luminal substances into the bloodstream and could lead to chronic inflammation with impairment of animal health and growth performances [5]. Furthermore, the increased intestinal permeability could induce bacterial translocation, and a systemic bacterial infection might also occur [6]. Therefore, protecting the intestinal tract from excessive oxidative damage and maintaining the integrity of the TJ could be major factors that positively influence the performance of birds in the intensive poultry industry [4,7].

Agricultural by-products, such as Wheat Bran (WB), contain Non-Starch Polysaccharides (NSPs) that act as anti-nutritional compounds in monogastric animals, which tend to inhibit digestibility and promote pathogen proliferation in the gastrointestinal tract, and eventually lead to gut inflammation and worsen the performance of the animals [8,9]. Solid-State Fermentation (SSF) could represent a valuable method to use agricultural by-products as substrates for NSP-degrading microorganisms, converting them into alternative feed ingredients [10,11]. In addition, filamentous fungi were reported as being a suitable inoculant for SSF due to their ability to withstand low humidity environments [11,12]. WB, a solid-state that is fermented by the *Trichoderma* sp., has been shown to exhibit antioxidant properties and to improve the nutrition value of WB, which could potentially be a low-cost feedstuff candidate [13,14]. Solid-state WB fermented by *Antrodia cinnamomea* was found to be suitable for producing bioactive compounds, such as phenolics, triterpenoids, and polysaccharides, as well as for growth promotion and its antioxidative and anti-inflammatory effects in broiler feeding trials [11,15]. According to these studies, the use of fungal SSF in agricultural by-products is viable for producing functional feedstuffs that contain bioactive compounds.

The *Laetiporus* sp. is a fungal species with medical properties, and it was traditionally used by Europeans to cure gastric cancer, rheumatism, pyretic diseases, and coughs [16]. In our previous studies, we showed that *L. sulphureus* fermented WB and potentially enhanced the growth performance of broilers by modifying their intestinal microflora and their immune status [17]. Furthermore, submerged mycelial cultures of *L. sulphureus* produce functional polysaccharides [18,19], as well as mycophenolic acids [20], and are able to dampen the excessive immune response of the selected cells without causing cytotoxicity [21,22]. Petrović et al. [23] reported that the aqueous extracts of wild *L. sulphureus* exert 1, 1-diphenyl-2-picrylhydrazyl (DPPH) radical scavenging activity, which correlates with its total phenolic content. Our previous study also indicated the potential of *L. sulphureus* to produce various bioactive compounds, such as crude phenolics, crude triterpenoids, polysaccharides, and ergosterol. The same study also showed that ethanol extracts of *L. sulphureus* fermented WB significantly attenuated the DNA damage induced by 2,2′-Azobis (2-amidinopropane) dihydrochloride (AAPH) in peripheral blood mononuclear cells of chickens, while exerting an in vitro antioxidant effect, including DPPH radical scavenging activity and reducing power [24]. However, the effects of *L. sulphureus* on the antioxidative status and TJ modulation in the gastrointestinal tracts of broilers has rarely been studied.

In order to evaluate the in vivo antioxidant properties of *L. sulphureus* solid-state fermented product (FL) in broiler chickens, nuclear factor erythroid 2–related factor 2 *(Nrf2)* and its downstream pathway was evaluated. Nrf2 is a redox-sensitive transcription factor that could be triggered by ROS, leading to the production of detoxification and antioxidant enzymes such as heme oxygenase-1 (HO-1), superoxide dismutase (SOD), catalase (CAT), and glutathione peroxidase (GPx) [2]. Therefore, this study was performed in order to evaluate if the in vitro antioxidant activity of FL could be used in vivo to improve the antioxidant status of broiler chickens and further investigated the effect of FL on broilers and the interaction between the antioxidation capacity and the expression of intestinal TJ mRNA.

## 2. Materials and Methods

### 2.1. Laetiporus sulphureus Culture, Inoculum Preparation, and Solid-State Fermentation

Methods for culturing *L. sulphureus* (Bull.) Murril and preparing FL were performed according to [17]. The *L. sulphureus* (Bull.) Murril used in this study was purchased from the Bioresource Collection and Research Center (BCRC, Hsinchu, Taiwan). The fungus was cultured on Malt Extract Agar (MEA) plates and incubated in a temperature-controlled incubator (PHCbi, Tokyo, Japan) at 25 °C, with a routine sub-cultivation (at a frequency of once per week). A solid-state fermentation inoculum was cultured in Erlenmeyer flasks containing 100 mL sterilized Malt Extract Broth (MEB). Five pieces of round-shaped agar (about 1 cm in diameter) were punched out from an *L. sulphureus* MEA plate and transferred to flasks containing 100 mL MEB. These flasks were incubated by a rotary shaker incubator (PHCbi, Tokyo, Japan) at 25 °C, at 120 rpm for five days (d). The malt extract broth that was grown with *L. sulphureus* filaments was put in a sterilized plastic bag and homogenized with a Seward Stomacher (Seward Laboratory Systems Inc., NY, USA) for the preparation of the inoculum. Solid-state fermentation was performed by adjusting 50 g of WB to 50% moisture content by adding distilled water and contained in a plastic bag. After autoclaved at 121 ± 1 °C for 30 min, the plastic bags containing sterilized WB were added with 10 mL of inoculants and fermented aerobically at 25 °C for 12 days. The fermented products were collected and dried at 40 °C for 2 days before being ground in a mill for the subsequent preparation of experimental broiler feed.

### 2.2. Experimental Birds and Housing

The animal experiment was authorized by the Animal Care and Use Committee of the National Chung Hsing University, Taiwan (IACUC No. 105-140). Four-hundred one-day-old male broilers (Ross 308) were randomly allocated into the five experimental diets: (1) corn-soybean meal (control), (2) a basal diet replaced with 5% WB (5% WB), (3) a basal diet replaced with 10% WB (10% WB), (4) a basal diet replaced with 5% FL (5% FL), and (5) a basal diet replaced with 10% FL (10% FL), with four replicates (pen)/diet and 20 birds per pen (a total of 80 birds/treatment). The replacement of WB or FL in the basal diet means the feed formula had 5% or 10% of the experimental ingredients, and the nutrient value of feeds in each control and treatment groups were adjusted to meet the nutrient requirements of broilers, as recommended by the National Research Council (NRC) [25], while each group had the same amount of ME and crude protein contents (Table 1 and Table 2). The experimental period included two phases: the starter phase (1–21 days) and the finisher phase (22–35 days), and provided to broilers without any anticoccidial or antibacterial supplements. The approximate composition, including crude protein, dry matter, and crude fat, was analyzed by following the method of the Association of Official Analytical Chemists (AOAC) [26] and demonstrated in our previous study [17] and shown in Table S1. At the start of the experiment, the average body weight of the birds was adjusted to make it uniform for all pens. The birds were kept in floor pens (2.5 × 4.0 m) with concrete floors and rice hull. The chicks were vaccinated against infectious bronchitis and Newcastle disease immediately after birth. The broilers had free access to feed and water during the whole experimental period. On day 35, the performance of the broilers was assessed by recording their Body Weight Gains (BWG), Body Weight (BW), Feed Intake (FI), and Feed Conversion Ratio (FCR; feed intake/body weight gain). The data of growth performances were demonstrated in Table 3 of our previous publication [17].

### 2.3. Collection of Serum, Intestinal Content, and Organs

On day 35, a total of six birds were randomly selected from a total of 80 birds per control or treatment group for sampling. Five mL blood samples were collected from the brachial vein and centrifuged at 2000× *g* for 10 min in order to collect the serum samples. Subsequently, the chickens were euthanized by exsanguination, and the organs (liver, jejunum, and ileum) were harvested and submerged in RNA shield^TM^ (ZYMO, Irvine, CA, USA) for mRNA isolation. All samples were stored at −80 °C until further analysis.

### 2.4. Determination of Serum Antioxidant Indexes

The collected serum from each treatment group was analyzed for the antioxidant indexes, including the CAT, superoxide dismutase (SOD), GPx activity, and glutathione (GSH), glutathione disulfide (GSSG), and malondialdehyde (MDA) levels. The serum CAT activity was determined by using the Cayman Chemical Catalase Assay Kit (Cayman Co, Ann Arbor, MI, USA, item No. 707002), based on the method of Wheeler et al. [27]. The SOD activity was detected by modifying the method of Wheeler et al. [27], using the Superoxide Dismutase Assay Kit (Cayman Co, USA, item No. 706002). The MDA concentration was tested according to the method described by Yagi [28] by using the 2-thiobarbituric acid reacting substances (TBARS) Assay Kit (Cayman Co, Ann Arbor, MI, USA, item No. 10009055). The Glutathione Peroxidase Assay Kit (Cayman Co, Ann Arbor, MI, USA, item No. 703102) was used for the examination of GPx activity, according to the method of Paglia et al. [29]. The serum levels of GSH and GSSG in 35-day-old chickens were assayed by using the glutathione kit (Cayman Co, Ann Arbor, MI, USA, item No. 703002), based on the method of Baker et al. [30].

### 2.5. RNA Extraction and Quantitative Reverse Transcription-Polymerase Chain Reaction

Organ samples submerged in the RNA shield ^TM^ (ZYMO, USA) were processed to extract RNA, by following the commercial kit manual (AllBio Science, Inc., Taichung, Taiwan). After the extraction, the concentrations of RNA samples were determined spectrophotometrically and diluted to 50 ng/μL. A quantitative real-time polymerase chain reaction (RT-qPCR) analysis was conducted by using the StepOnePlus ™ Real-Time PCR System (Roche Diagnostics Ltd., Taipei, Taiwan), according to the method of Lin et al. [31]. The primers that were applied in the experiment are provided in Table 3, based on the genes of *Gallus gallus*. The data of the gene expressions from the same treatment were normalized to β-actin (housekeeping gene). The means and Standard Deviation (SD) were calculated.

### 2.6. Intestinal Morphology Evaluation

The jejunum (from the pancreatic loop to Meckel’s diverticulum) and ileum (from Meckel’s diverticulum to the ileo-caeco-colic junction) samples from each bird (approximately 3 cm) were collected and fixed in 10% formalin. After washing them in a phosphate buffer saline solution and including them in paraffin wax, samples were cut at a thickness of 3 μm and stained with hematoxylin and eosin. The Motic image plus 2.0 (Motic Inc., Schertz, TX, USA), combined with light microscopy, was applied for the measurement of the villus height and crypt depth. Twenty spots of villi and crypt from the intestinal samples from six birds in each control and treatment groups were measured and calculated for the ratio of the villus height to the crypt depth.

### 2.7. Statistical Analysis

Data were subjected to analysis of variance (ANOVA) as a completely randomized design by using the generalized linear model (GLM) function in the SAS software (SAS 9.4, 2018). Normality of data distribution was checked, significant statistical differences were determined among the various treatment group means, using Tukey’s honest significant difference test. The effects of the experimental diet on the response variables were considered to be significant at *p* < 0.05.

## 3. Results

### 3.1. Growth Performance

The growth performances of one- to 35-day-old broilers were demonstrated in Table 3 of our previous publication [17]. No significant difference was observed in the body weight, body weight gain, and feed intake of each group of 1–35 day-old broilers and 1–21 day-old broilers (*p* > 0.05). While 5% FL group had significantly higher BW than the control group and 10% WB group during 22–35 d. The FCR of 5% and 10% FL supplemented groups (both 1.41) were significantly lower than that in the control group (1.47), as well as in the 5% and 10% WB groups (1.45 and 1.46, respectively) (*p* < 0.05).

### 3.2. Serum Antioxidant Profiles

The serum antioxidant profiles of 35-day-old broilers supplemented with FL are presented in Table 4. Compared to the control group, 5% and 10% WB group, the 5% FL supplementation significantly improved SOD activity, while the concentration of MDA in the serum was down-regulated (*p* < 0.05). However, there was no significant difference in the indexes, including the CAT activity, GPx activity, GSH concentration, GSSG concentration, and GSH:GSSG (*p* > 0.05).

### 3.3. Expression of Selected Antioxidant Genes

**Figure 1:**
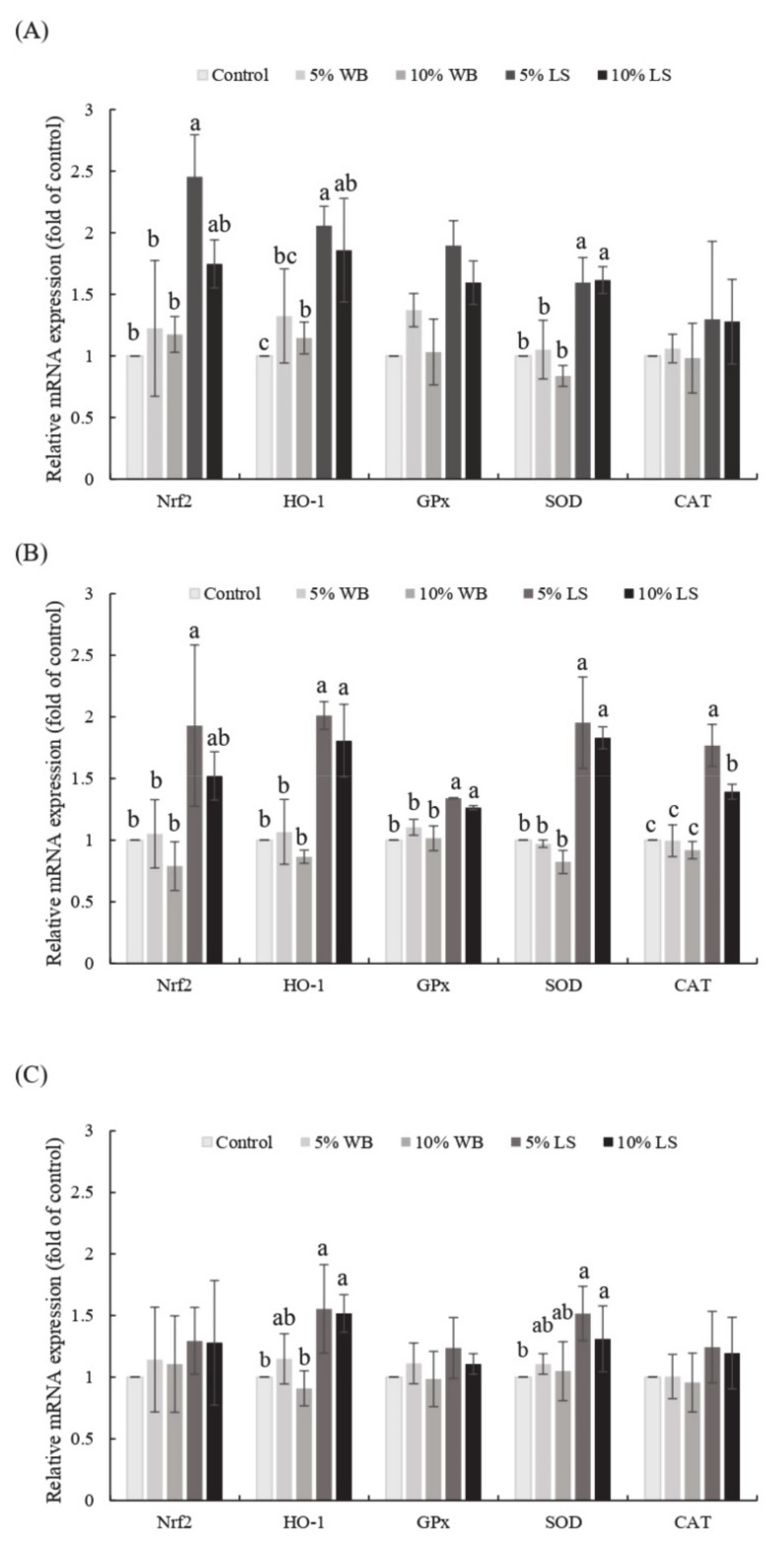
Effects of FL supplementation on mRNA expression levels of the selected genes related to antioxidant-status in (**A**) liver, (**B**) jejunum, and (**C**) ileum of 35-day-old broilers. Values are expressed as the mean ± standard deviation (*n* = 6). ^a–c^ Means among groups without the same letter, within the same sampling day, are significantly different (*p* < 0.05). WB: Wheat Bran; FL: *Laetiporus sulphureus* fermented product.

The expression profile of selected antioxidant genes in the liver, jejunum, and ileum are displayed in Figure 1. Both FL groups had a significantly elevated *HO-1* and *SOD* expression in the liver, jejunum, and ileum, compared to those in the control group (*p* < 0.05). Furthermore, the 5% FL group had a significantly elevated *HO-1* and *Nrf2* expression in the liver and jejunum, compared to those in WB and control groups (*p* < 0.05). In addition, the 5% FL supplementation group had a significantly elevated expression of *Nrf2* in the jejunum, compared to the control and WB groups (*p* < 0.05). In the liver, there was no significant change in the *CAT* and *GPx* mRNA expression of each group (*p* > 0.05), while no significant differences between 10% FL vs. 5% WB and 10% WB as well as 5% FL vs. 10% FL in the relative mRNA expression of *HO-1*. The FL groups had a significantly better expression of *GPx* and *CAT*, compared to those in the jejunum of the WB and control groups (*p* < 0.05). However, there was no significant difference among each group for *Nrf2*, *GPx*, and *CAT* expression in the ileum, while mRNA expression of *HO-1* in both FL groups was not significantly different from the 5% WB group (*p* > 0.05). Both FL groups showed no significant difference in the mRNA expression of *SOD* compared to the WB groups in the ileum (*p* > 0.05).

### 3.4. Selected Tight Junction (TJ) Gene Expression

**Figure 2:**
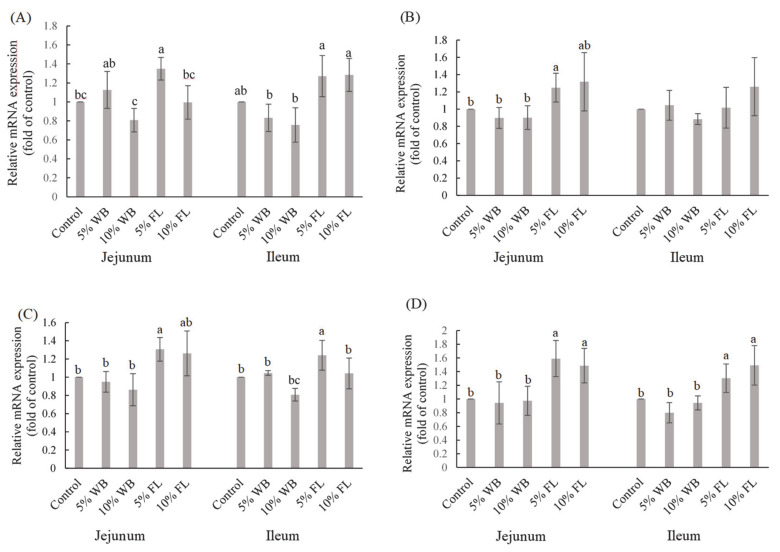
The effects of FL supplementation on *ZO-1* (**A**), *CLDN-1* (**B**), *MUC-2* (**C**), and *OCLN* (**D**) mRNA expression in the jejunum and ileum of 35-d-old broiler chickens. Values are expressed as the mean ± standard deviation (*n* = 6). ^a–c^ Means within the same rows, without the same superscript letter, are significantly different (*p* < 0.05). WB: Wheat Bran; FL: *Laetiporus sulphureus* fermented product; *ZO-1*: Zonula occludens-1; *CLDN-1*: Claudin-1: *MUC-2*: Mucin*-*2; *OCLN*: Occludin.

The effects of FL on selected TJ mRNA expressions are presented in Figure 2. Wheat bran supplementation in broilers had no significant effect on the expression of selected TJ mRNA in both the jejunum and the ileum (*p* > 0.05). Tight junction mRNA expressions in the jejunum showed that the 5% FL group had significantly elevated *CLDN-1* expression, compared to the control and WB groups, while both FL supplementations significantly improved the expression of *OCLN* in the jejunum and ileum (*p* < 0.05). A significantly higher *MUC-2* expression in the jejunum and ileum was observed only in the 5% FL supplemented group, compared to that in the control group (*p* < 0.05). In addition, 5% and 10% FL supplementation significantly improved the expression of *ZO-1* in jejunum and ileum, compared to that in the 5% and 10% WB groups (*p* < 0.05). However, there were no significant differences among the groups in terms of the ileal expression of *CLDN-1* (*p* > 0.05).

### 3.5. Intestinal Morphology

**Figure 3:**
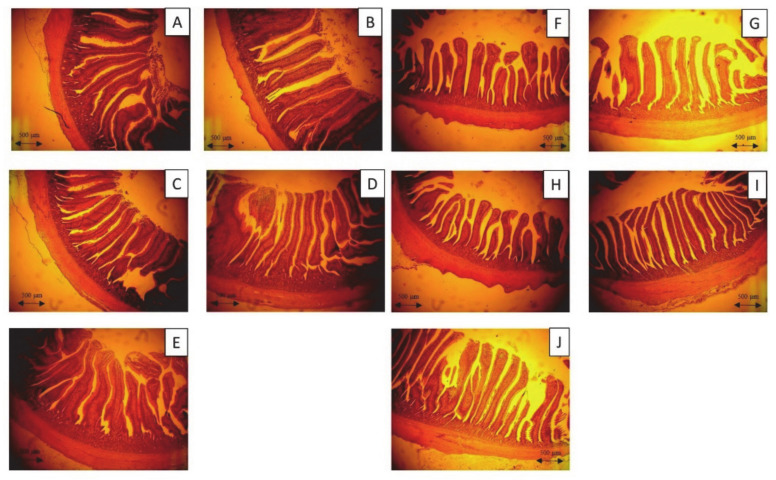
Photomicrography of the jejunum and ileum of 35-day-old broiler fed with control diet and *Laetiporus sulphureus* fermented product. (**A**)–(**E**)—the jejunum of 35-day-old broilers representing the control, 5% WB, 10% WB, 5% FL, and 10% FL groups, respectively. (**F**)–(**J**)—the ileum of 35-day-old broilers representing the control, 5% WB, 10% WB, 5% FL, and 10% FL groups, respectively. Haematoxylin and eosin stain (40×).

The changes in intestinal morphology of 35-day-old broilers in each control and treatment group are listed in Table 5. The results show that 5% FL supplementation significantly increased the villus height in the ileum and jejunum, compared to those in the WB and control groups, while both FL supplementation groups had significantly higher villus in the jejunum, compared to the 10% WB group (*p* < 0.05). Furthermore, the jejunal and ileal crypt depth in the 10% FL group was significantly reduced compared to that of the control group (*p* < 0.05). A significantly higher villi:crypt ratio in the ileum was observed in the FL groups, compared to that in the WB and control groups (*p* < 0.05). However, the villi:crypt ratio in the jejunum of both FL groups was not significantly different from that in the control and WB groups (*p* > 0.05). The microscopic images of the jejunum and ileum sections are displayed in Figure 3.

## 4. Discussion

In order to evaluate the in vivo antioxidant properties of FL in broiler chickens, we focused on *Nrf2* and its downstream pathway. In this study, the expression of *Nrf2*, *HO-1*, and *SOD* mRNAs in FL supplemented groups were up-regulated in the liver and jejunum, compared to those in the control group, which indicates the antioxidant role of FL in broilers. Nrf2 is a redox-sensitive transcription factor that is localized in the cytoplasm and binds with Kelch-like ECH-associated protein 1 (Keap 1), an actin-binding protein, under normal conditions [32]. Upon activation by ROS, Nrf2 dissociates from Keap 1, interacts with the antioxidant response elements, and regulates the expression of downstream antioxidant genes to activate the antioxidant and detoxifying effects [2]. The induction of *HO-1* is an important cellular process for dealing with oxidative stress by degrading the intracellular levels of pro-oxidant heme and by producing biliverdin (a precursor of bilirubin) [33]. The elevated antioxidative status of FL supplemented groups could be due to the bioactive phenolic compounds within FL. The phenolic compounds derived from fungus have been reported to activate the Nrf2-Keap1 pathway in a suppressed *Nrf2* diabetic rat model [34]. Furthermore, *A. cinnamomea*, a brown-rot fungus that exerted medicinal effects similar to those of *L. sulphureus*, reported the enhanced expression of *Nrf2*, *HO-1*, and *SOD* mRNAs in the liver of 35-day-old chickens fed upon its fermented feedstuff, as well as a further increase in SOD activity in the serum [11,15]. Therefore, it can be validated that FL, as a medicinal fungus fermented products, may have also improved the antioxidative status of broilers via a similar mechanism as reported by above mentioned previous studies.

Non-enzymatic and enzymatic antioxidant systems are two strategies that are used in cells to inhibit the potential ROS toxicity. Enzymatic antioxidants are endogenously synthesized and regulated, which is a crucial indicator for evaluating the oxidative status of animal tissues [11,15]. SOD is an endogenous antioxidant enzyme that catalyzes the dismutation of O^2-^ to H_2_O_2_ and O_2_. In our study, the elevated SOD activity in the 5% FL supplemented group indicated an increase in the extracellular antioxidant enzymatic activity. Similarly, Lin et al. [35] observed that the dietary supplementation of mulberry leaves improved the serum SOD levels and showed that these outcomes were due to the presence of abundant phenolic compounds in the tested product, which was in common with FL that consisted of phenolic compounds that exerted antioxidant ability. The development of oxidative injury could be indicated by the serum concentration of MDA because it is one of the end products of lipid peroxidation [36]. In this study, the FL-supplemented groups had a higher SOD activity and a lower MDA concentration. The elevated expression of serum SOD and *HO-1* mRNA enhanced the capacity of broilers to catalyze the harmful radicals and encounter potential oxidative damage, which further led to the reduction of MDA in the serum, which represents a total decline in the lipid oxidation in animals. Likewise, Lee et al. [11] reported that the *A. cinnamomea-*fermented product supported the antioxidant status of broiler chickens by improving the SOD activities. Furthermore, the enzyme powder fermented by *Trichoderma pseudokoningii* was also found to exert an antioxidant effect on broiler chickens, which increased the serum SOD activities and reduced the MDA concentration [14]. Interestingly, FL seemed to have a less-pronounced effect on the ileum, which might be due to the direct absorbance of a simple-structured polyphenol in the jejunum, while the remaining complicated polyphenols are more likely to be fermented in the hindgut by cecal microbiota and to exert their bio-functional effects [37].

According to the results of this study, the increased mRNA expression of transmembrane proteins claudin-1 (CLDN-1), occludin (OCLN), and mRNA of peripheral membrane protein zonula occluden-1 (ZO-1) may be caused by the enhancement of the antioxidative status in the jejunum of the FL-supplemented groups. By improving the function of TJ and eliminating the deleterious effects of oxidative stress, the increase in *MUC-2* mRNA expression could lead to the improved health condition of intestinal goblet cells. The integrity of epithelial cells and the normal function of the intestine in broiler chickens is maintained by the TJs, which consist of several crucial elements, including occludin, claudins, and ZOs. *OCLN* translates to occludin that forms the TJs, and its sealing property is involved in the hurdle functions of the epithelial barrier [38]. Claudins (translated from *CLDN*) are another family of integral membrane proteins that collaborate with occludin to maintain the integrity of the TJs [5]. ZO-1 and ZO-2 bind directly to the COOH terminus of the intracellular domain of occludin, which contributes to the normal structure of the epithelial barrier function [6]. In addition, the *MUC-2* gene is expressed by goblet cells, which produce a mucus layer that helps in blocking pathogens invasion along with TJs [39]. The TJs could be disturbed when animals encounter heat stress and oxidative damage due to excessive free radicals, which could lead to leaky gut and onset of inflammatory response with poor animal health and impaired growth performances as a consequence. Moreover, *MUC-2* deficiency in mice causes spontaneous inflammation and allows the colonization of unusual commensal bacteria [40]. The interaction of the antioxidant status and TJ integrity has also been proven in several studies by using various animals and cell models. Chen et al. [9] reported that riboflavin deprivation decreased the antioxidant enzyme activities, such as SOD, GPx, and glutathione reductase activities in young grass carp, which further reduced the expression of TJ mRNA, *OCLN*, and *ZO-1*. Zhao et al. [41] reported that the *MUC-2* function could be interrupted by bisphenol A, which induces mitochondrial dysfunction and oxidative stress. Exogenous antioxidants, including polyphenols and polysaccharides, help to scavenge the excessive free radicals and prevent oxidative damage to the intestinal cells and proteins [42]. In an H_2_O_2_-induced oxidative damaged Caco-2 cell model, a phytogenic called red-osier dogwood, which contained abundant phenolic compounds, was introduced to enhance the expression of *HO-1*, *SOD*, *GPx* genes, and the Nrf2 protein, while improving the cell-integrity by increasing the expression of ZO-1 and claudin-3 that were damaged by H_2_O_2_. In addition, Sun et al. [43] demonstrated that the essential oil cinnamaldehyde exerted an antioxidant activity and elevated the protein expression of claudin-4, occluding, and ZO-1. Chitosan oligosaccharide, an antioxidant and an immunomodulatory substance, has been proven to improve *OCLN* mRNA expression in the jejunum of broilers, while an increase in antioxidant enzymes and reduced levels of proinflammatory cytokine IL-6 were also observed [44]. Quercetin, an antioxidant flavonoid, was demonstrated to increase *MUC-2* gene expression in human intestinal goblet cell-like LS174T [45]. Therefore, the improvement of the *MUC-2* status in broilers supplemented with FL could result in the elevation of TJ integrity.

The villus height and crypt depth, with respect to the morphologies of the jejunum and ileum of broilers, are always used as an indicator of intestinal health. The villus height represents nutrient absorption efficiency, and a low crypt depth is favored, due to the reduced intestinal cell turnover rate, to save energy and achieve a better growth performance [46,47]. In our previous study, lignocellulose enzymes (laccase and xylanase), which are capable of degrading the deleterious effects of NSP-rich WB, were produced during *L. sulphureus* fermentation [17]. The improved morphologies of the jejunum and ileum in broilers could be due to the lignocellulose in FL. Similar results were also demonstrated by Chu et al. [13], who observed a positive impact of *Trichoderma* fermented wheat bran on the intestinal morphology of broilers, while Lin et al. [14] showed that the *T. pseudokoningii* fermented enzyme powder that contains NSPase improved the intestinal morphology of broilers. Furthermore, FL was found to be rich in phenolic compounds and capable of exerting an antioxidant effect, thus preventing oxidative stress. In the lumen of the intestine, stressors, including pathogens and pro-oxidants, can cause dynamic changes in the intestinal mucosa due to the close proximity of the mucosal surface and the intestinal contents [1]. According to the outcomes of TJ and intestinal morphology, the antioxidant effect of FL could potentially protect the intestinal mucosa, as well as the integrity of epithelial cells, from oxidative damage. Similar results were shown by Viveros et al. [48], who suggested that the dietary grape pomace concentrate (60 g/kg in feed) that are rich in polyphenols could improve the ratio of villus height and crypt depth (villus height: crypt depth) and increased the feed efficiency of broilers. Likewise, Lai et al. [8] reported that fermentation of soybean hulls containing phenolic-rich residues of *Pleurotus eryngii* stalk increased the villus height/crypt depth in the ileum. In this study, FL improved the villi:crypt ratio in the ileum compared to the control and WB groups, while higher villi and less shallow crypts were discovered in both jejunum and ileum of 5% FL group compared to the WB and control groups. These improvements in intestinal morphology could be possibly caused by the antioxidant effect along with the potential existence of NSP enzymes.

## 5. Conclusions

In conclusion, FL is capable of improving the FCR in broilers by improving the antioxidative status of broilers while and enhancing intestinal TJ mRNA expression while minimizing the negative effects of dietary NSP. In addition, 5% FL supplementation had the best overall results in enhancing the serum SOD activity and antioxidant gene expression in the liver, jejunum, and ileum while improving the intestinal TJ mRNA expression.

## Figures and Tables

**Table 1:** Ingredients of the experimental diets for broilers at the starter phase (days 1–21).

Ingredients	Starter Phase (Days 1–21)				
	Control	5% WB	5% FL	10% WB	10% FL
	g/kg				
Corn	524.9	458.7	458.4	392.5	391.9
WB	0	50.0	0	100.0	0
FL	0	0	50.0	0	100.0
Soybean meal, CP 44%	320.0	167.3	167.3	14.8	14.7
Fish meal, CP 60%	50.0	50.0	50.0	50.0	50.0
Full fat soybean meal	41.4	209.8	210.1	377.9	378.7
Soybean oil	30.0	30.0	30.0	30.0	30.0
Limestone	11.6	11.6	11.6	11.5	11.5
Monocalcium phosphate	11.2	11.2	11.2	11.2	11.2
_DL_-Methionine	3.4	3.7	3.7	4.1	4.1
Sodium chloride	2.9	2.8	2.8	2.8	2.8
_L_-Lysine HCl	1.8	2.1	2.1	2.4	2.3
Choline-Cl (60%)	0.8	0.8	0.8	0.8	0.8
Vitamin premix ^1^	1.0	1.0	1.0	1.0	1.0
Mineral premix ^2^	1.0	1.0	1.0	1.0	1.0
Total	1000.0	1000.0	1000.0	1000.0	1000.0
Calculated nutrient value					
ME, kcal/kg	3050.0	3050.0	3050.0	3050.0	3050.0
Dry matter, %	88.28	88.85	89.11	89.42	89.95
Crude protein, %	23.0	23.0	23.0	23.0	23.0
Crude fat, %	6.04	8.86	8.64	11.68	11.25
Calcium, %	1.05	1.05	1.05	1.05	1.05
Total phosphorus, %	0.73	0.73	0.73	0.72	0.72
Available phosphorus, %	0.50	0.50	0.50	0.50	0.50
Lysine, %	1.43	1.43	1.43	1.43	1.43
Methionine, %	0.73	0.74	0.74	0.76	0.76
Cysteine, %	0.34	0.32	0.32	0.31	0.31

WB: wheat bran; FL: *Laetiporus sulphureus* fermented wheat bran. ^1^ Supplied per kg of diet: Vit. A, 15000 U; Vit. D3, 3000 U; Vit. E, 30 mg; Vit. K3, 4 mg; Riboflavin, 8 mg; Pyridoxine, 5 mg; Vit. B12, 25 μg; Ca-pantothenate, 19 mg; Niacin, 50 mg; Folic acid, 1.5 mg; Biotin, 60 μg. ^2^ Supplied per kg of diet: Co (CoCO_3_), 0.255 mg; Cu (CuSO_4_·5 H_2_O), 10.8 mg; Fe (FeSO_4_·H_2_O), 90 mg; Zn (ZnO), 68.4 mg; Mn (MnSO_4_·H_2_O), 90mg; Se (Na_2_SeO_3_), 0.18 mg.

**Table 2:** Ingredients of the experimental diets for broilers at the finisher phase (days 22–35).

Ingredients	Finisher Phase (Days 22–35)				
	Control	5% WB	5% FL	10% WB	10% FL
	g/kg				
Corn	549.5	482.9	482.9	416.3	416.3
WB	0	50.0	0	100.0	0
FL	0	0	50.0	0	100.0
Soybean meal, CP 44%	16.6	185.6	185.6	354.4	354.4
Fish meal, CP 60%	320.6	167.8	167.8	15.1	15.1
Full fat soybean meal	30.0	30.0	30.0	30.0	30.0
Soybean oil	10.6	10.6	10.6	10.6	10.6
Limestone	12.2	12.2	12.2	12.2	12.2
Monocalcium phosphate	3.4	3.3	3.3	3.2	3.2
_DL_-Methionine	50.0	50.0	50.0	50.0	50.0
Sodium chloride	1.3	1.5	1.5	1.8	1.8
_L_-Lysine HCl	3.0	3.3	3.3	3.6	3.6
Choline-Cl (60%)	0.8	0.8	0.8	0.8	0.8
Vitamin premix ^1^	1.0	1.0	1.0	1.0	1.0
Mineral premix ^2^	1.0	1.0	1.0	1.0	1.0
Total	1000.0	1000.0	1000.0	1000.0	1000.0
Calculated nutrient value					
ME, kcal/kg	3175.0	3175.0	3175.0	3175.0	3175.0
Dry matter, %	88.31	88.88	89.15	89.45	89.98
Crude protein, %	21.0	21.0	21.0	21.0	21.0
Crude fat, %	7.56	10.39	10.17	13.22	12.78
Calcium, %	0.90	0.90	0.90	0.90	0.90
Total phosphorus, %	0.68	0.67	0.67	0.67	0.67
Available phosphorus, %	0.45	0.45	0.45	0.45	0.45
Lysine, %	1.25	1.25	1.25	1.25	1.25
Methionine, %	0.65	0.66	0.66	0.67	0.67
Cysteine, %	0.31	0.30	0.30	0.29	0.29

WB: wheat bran; FL: *Laetiporus sulphureus* fermented product. ^1^Supplied per kg of diet: Vit. A, 15000 U; Vit. D3, 3000 U; Vit. E, 30 mg; Vit. K3, 4 mg; Riboflavin, 8 mg; Pyridoxine, 5 mg; Vit. B12, 25 μg; Ca-pantothenate, 19 mg; Niacin, 50 mg; Folic acid, 1.5 mg; Biotin, 60 μg. ^2^Supplied per kg of diet: Co (CoCO_3_), 0.255 mg; Cu (CuSO_4_·5 H_2_O), 10.8 mg; Fe (FeSO_4_·H_2_O), 90 mg; Zn (ZnO), 68.4 mg; Mn (MnSO_4_·H_2_O), 90mg; Se (Na_2_SeO_3_), 0.18 mg.

**Table 3:** Characteristrics and performance data of the primers used for RT-qPCR analysis.

Genes	Forward Primer (from 5′ to 3′)	NCBI GenBank
	Reverse Primer (from 5′ to 3′)	
*β*-actin	CTGGCACCTAGCACAATGAA	X00182.1
	ACATCTGCTGGAAGGTGGAC	
*HO-1*	AGCTTCGCACAAGGAGTGTT	X56201.1
	GGAGAGGTGGTCAGCATGTC	
*SOD*	GCCACCTACGTGAACAACCT	NM_204211.1
	AGTCACGTTTGATGGCTTCC	
*Nrf2*	GGAAGAAGGTGCTTTTCGGAGC	NM_205117.1
	GGGCAAGGCAGATCTCTTCCAA	
*CAT*	CCACGTGGACCTCTTCTTGT	NM_001031215.1
	AAACACTTTCGCCTTGCAGT	
*GPx*	CAGCAAGAACCAGACACCAA	NM_001163245.1
	CCAGGTTGGTTCTTCTCCAG	
*ZO-1*	AGGTGAAGTGTTTCGGGTTG	XM_015278975.1
	CCTCCTGCTGTCTTTGGAAG	
*CLDN-1*	GGAGGATGACCAGGTGAAGA	NM_001013611.2
	TCTGGTGTTAACGGGTGTGA	
*MUC-2*	GCTACAGGATCTGCCTTTGC	NM_001318434.1
	AATGGGCCCTCTGAGTTTTT	
*OCLN*	GTCTGTGGGTTCCTCATCGT	NM_205128.1
	GTTCTTCACCCACTCCTCCA	

RT-qPCR: real-time quantitative polymerase chain reaction; NCBI: National Center for Biotechnology Information; *HO-1*: Heme oxygenase -1; *SOD*: Superoxide dismutase, mitochondrial; *Nrf2*: Nuclear factor (erythroid-derived 2)-like 2; *CAT*: catalase; *GPx*: Glutathione peroxidase; *ZO-1*: Zonula occludens-1; *CLDN-1*: Claudin-1: *MUC-2*: Mucin 2; *OCLN*: Occludin.

**Table 4:** Effects of FL supplementation on serum antioxidant profiles in 35-day-old broilers ^1^.

Items	Treatments					SEM	*p-*Values
	Control	5% WB	10% WB	5% FL	10% FL		
SOD (U/mL)	9.18 ^bc^	9.36 ^b^	8.96 ^c^	9.82 ^a^	9.40 ^b^	0.05	0.001
CAT (U/mL)	15.35	15.82	15.57	15.81	15.53	0.11	0.86
MDA (μM)	28.52 ^a^	25.76 ^b^	29.66 ^a^	23.53 ^c^	24.17 ^bc^	0.21	<0.0001
GPx (U/mL)	146.42 ^ab^	148.14 ^a^	141.51 ^b^	151.66 ^a^	151.13 ^a^	0.84	0.005
GSH (mg/mL)	2.01	2.17	2.13	2.21	2.19	0.04	0.71
GSSG (mg/mL)	0.11	0.12	0.14	0.13	0.11	0.005	0.72
GSH:GSSG	20.65	19.14	16.52	21.08	21.71	0.65	0.51

^1^ The results are expressed as the means of six replicates in each control and treatment group. SEM: Standard Error of the Mean. WB: Wheat Bran; FL: *Laetiporus sulphureus* fermented product; SOD: Superoxide dismutase; CAT: catalase; MDA: malondialdehyde; GPx: glutathionine peroxidase; GSH: Glutathione; GSSG: oxidized glutathione. ^a–c^ Means within the same rows, but without the same superscript letter, are significantly different (*p* < 0.05).

**Table 5:** Effects of FL supplementation on intestinal morphology in 35-day-old broilers ^1.^

Items	Treatments					SEM	*p*-Values
	Control	5% WB	10% WB	5% FL	10% FL		
Jejunum							
Villus height (μm)	1210.72 ^bc^	1207.34 ^bc^	1157.55 ^c^	1325.91 ^a^	1255.74 ^ab^	10.29	0.001
Crypt depth	270.72 ^a^	203.38 ^c^	216.52 ^bc^	244.50 ^ab^	212.41 ^bc^	5.28	0.002
Villi:crypt ratio	5.10	5.98	5.52	5.78	5.96	0.12	0.16
Ileum							
Villus height	1058.68 ^b^	1118.71 ^b^	1032.24 ^b^	1126.53 ^a^	113047 ^a^	8.10	0.04
Crypt depth	198.27 ^a^	190.01 ^ab^	188.03 ^ab^	178.80 ^ab^	170.71 ^b^	2.23	0.04
Villi:crypt ratio	5.98 ^b^	5.99 ^b^	5.51 ^b^	6.50 ^a^	6.24 ^a^	0.07	0.03

^1^ The results are presented as the means of twenty spots corresponding to six birds each for the control and treatment groups. WB: Wheat Bran; FL: *Laetiporus sulphureus* fermented product; SEM: standard error of the mean. ^a–c^ Means within the same rows, without the same superscript letter, are significantly different (*p* < 0.05).

## Data Availability

The data presented in this study are openly available at 10.1016/j.psj.2020.04.011., reference number [17].

## Supplementary Materials

The following are available online at https://www.mdpi.com/2076-2615/11/1/149/s1, Table S1: Chemical composition of the experimental diets for broilers.

## Abbreviations

WB	Wheat bran
FL	*L. sulphureus* fermented product
TJ	Tight junction
NSPs	Non-starch polysaccharides
MEB	Malt extract broth
MEA	Malt extract agar
RT	Room temperature
DPPH	1,1-diphenyl-2-picrylhydrazyl
BHT	Butylated hydroxytoluene
chPBMC	Chicken peripheral blood mononuclear cells
AAPH	2,2′-Azobis (2-amidinopropane) dihydrochloride
LPS	Lipopolysaccharide
FCR	Feed conversion ratio
SOD	Superoxide dismutase
CAT	Catalase
MDA	Malondialdehyde
GPx	Glutathionine peroxidase
GSH	Glutathione
GSSG	Glutathione disulfide
HO-1	Heme oxygenase -1
Nrf2	Nuclear factor (erythroid-derived 2)-like 2
CAT	Catalase
GPx	Glutathione peroxidase
ZO-1	Zonula occludens-1
CLDN-1	Claudin-1
MUC-2	Mucin-2
OCLN	Occludin
