# Supplementary material for: The Laetiporus sulphureus Fermented Product Enhances the Antioxidant Status, Intestinal Tight Junction, and Morphology of Broiler Chickens
Source: Animals (Basel). 2021 Jan 11;11(1):149. doi: 10.3390/ani11010149 (PMC7827109; doi:10.3390/ani11010149)
Supplement: Supplementary file 1 [file animals-11-00149-s001.pdf]

Table S1. Chemical composition of the experimental diets for broilers.

Analyzed Nutrient Value	Treatments				
	Control	5% WB	5% FL	10% WB	10% FL
**Starter phase (1–21 days)**					
Dry matter, %	88.22	88.67	89.11	89.05	89.95
Crude protein, % DM	23.54	23.48	23.45	23.52	23.40
Crude fat, % DM	6.32	8.89	8.63	11.44	11.23
**Finisher phase (22–35 days)**					
Dry matter, %	88.71	88.79	89.04	89.20	89.48
Crude protein, % DM	21.83	21.77	21.67	21.25	21.24
Crude fat, % DM	7.23	10.33	10.73	12.49	12.74

WB: wheat bran; FL: *Laetiporus sulphureus* fermented product; DM: dry matter.
